# Barriers and facilitators to physical activity and further digital exercise intervention among inactive British adolescents in secondary schools: a qualitative study with physical education teachers

**DOI:** 10.3389/fpubh.2023.1193669

**Published:** 2023-06-02

**Authors:** Richard Moore, Lee Edmondson, Maxine Gregory, Kerry Griffiths, Elizabeth Freeman

**Affiliations:** ^1^Sport and Physical Activity Research Centre, Sheffield Hallam University, Sheffield, United Kingdom; ^2^Department of Psychology, Sociology and Politics, Sheffield Hallam University, Sheffield, United Kingdom

**Keywords:** adolescents, PE teachers, COM-B model, physical activity, digital exercise intervention, United Kingdom, secondary schools

## Abstract

**Background:**

Previous studies indicated that physical education programs in schools were unsuccessful to ameliorate physical activity (PA) behaviors among adolescents. This study investigated PE teachers’ perceptions of barriers and facilitators to PA and further digital exercise interventions among inactive British adolescents in secondary schools based on the Capability, Opportunity, Motivation, and Behavior (COM-B) model, the Behavior Change Wheel (BCW), and Theoretical Domains Framework (TDF).

**Method:**

A qualitative study was conducted among 156 PE teachers in England. deductive thematic analysis approach was applied to analyze data.

**Results:**

A comprehensive perception of PE teachers revealed 21 barriers to PA among inactive adolescents in secondary schools. The study findings show that barriers exist across all categories of the COM-B model in physical opportunity (7), reflective motivation (5), social opportunity (4), psychological capability (4) and physical capability (1). The majority of these barriers were reported in previous studies as being barriers to PA from the perspective of children and adolescents. This shows that the findings are consistent with the views of children and adolescents that participated in these studies. Particular salient barriers for inactive adolescents were reported and greater insight into their experiences was highlighted. The study reported the main sources of behavior, intervention functions, policy functions, and behavior change tools that can be used for future behavior change interventions to support inactive adolescents.

**Conclusion:**

The study recommends using its findings to design interventions for inactive adolescents to achieve recommended levels of physical activity (PA). The study’s comprehensive approach and evidence-based solutions provide extensive reference points for future intervention design, informing policy and contributing to enhancing support for inactive adolescents. Further development of digital exercise interventions, including conversational artificial intelligence (AI), is suggested to engage adolescents at scale and provide personalized support to overcome multiple barriers to PA.

## 1. Introduction

Secondary schools play a critical role in promoting and guiding adolescents toward health-promoting activities. This includes encouraging and supporting physically inactive adolescents to become physically active through physical education (PE) and extra-curricular activities. Provision of sports and PE opportunities is neccessary to develop the physical literacy of adolescents and promote lifelong physical activity (PA) behaviors. In addition to fostering enjoyment and social interaction, participation in physical activities can lead to enhanced educational attainment and improved health outcomes for students (1–3). However, a worldwide umbrella study has found that efforts to promote PA in schools have mostly been unsuccessful in changing PA behaviors (4). The success of school-based interventions can be hindered by various factors, including poor implementation, which is partly the responsibility of PE teachers (4, 5).

Numerous studies have investigated why some people are more active than others (6). However, there is a limited understanding of the attitudes and behaviors of inactive adolescents who are more susceptible to the negative impacts of physical inactivity. PE teachers play a fundamental role in adolescents’ lives, by promoting and encouraging them to be physically active, yet there is inadequate knowledge of their perspectives on inactive adolescents. Several interventions have been implemented worldwide to tackle inactivity yielding mixed results (7). By gaining a deeper understanding of the barriers that PE teachers perceive, it is possible to develop future approaches and identify solutions that would benefit inactive adolescents.

In recent years, there has been a paradigm shift in the design of PA interventions. Rather than focusing solely on individual factors, PA interventions are now being designed to reshape the entire system, encompassing policy, environmental, social, and individual factors. This approach commonly known as a ‘whole systems approach’, involves applying systems thinking, to identify solutions that may involve multiple stakeholders in addressing the challenges of physical inactivity (8). Thus, multicomponent interventions are now favored over single-component interventions. The World Health Organization (WHO) used a systems framework to develop their Global Action Plan on PA, with four strategic objectives, and twenty policy actions aimed at reducing physical inactivity by 10% globally (9). Two of these objectives include enhancing PE and school-based programs in educational settings and prioritizing programs for the inactive (9). Schools and PE teachers play a fundamental role in this system, as they are responsible for supporting and engaging adolescents in PA every week throughout the school term. Therefore, they have an unrivaled level of understanding of the barriers and facilitators that exist for this population.

According to the PA guidelines set by the Chief Medical Officer of the UK, it is recommend that adolescents engage in moderate to vigorous intensity PA for at least 60 min every day (10). However, in England, around 2.2 million adolescents which accounts for 30% of the population, are considered ‘less active’ (11) as they engage in less than 30 min of moderate to vigorous PA per day. To combat this, schools play a crucial role in encouraging PA as the UK Government recommends that at least 30 min of daily PA should take place within schools (i.e., PE and extracurricular activity), while the remaining 30 min should be completed outside of school time.

Previous research has shown that school-based physical education can play an important role in promoting daily moderate to vigorous PA and reducing sedentary behavior among adolescents, particularly those who are inactive (12–14). However, in the UK there has been a decline in the number of adolescents who meet the minimum recommended PA target per day, and a decrease in the duration of PE offered to adolescents as they transition through school. An analysis of data from the 2018/19 Active Lives Children and Young People survey revealed that 40% of adolescents engaged in 30 min or more of PA every day at school, while 57% did so outside of school (15). As a result, adolescents are more likely to be less active at school (60%) than they are outside of school (43%). Consequently, many schools are failing to enable adolescents to meet the minimum recommended PA target, thus contributing to their inactivity. Other challenges, such as low-quality PE and a lack of teachers’ commitment, have been reported in other countries, which may be contributing to global inactivity levels (4, 16).

It was reported in a systematic review that the main barriers and facilitators to the implementation of PA policies in schools are the ‘environmental context and resources’ such as the availability of equipment, time, or staff; ‘goals’ such as the perceived priority of the policy in the school; ‘social influences’, such as support from school boards; and ‘skills’ such as teachers’ ability to implement the policy. While previous studies have identified PA preferences and barriers (17–19) among adolescents in schools, there is a lack of evidence on the habits and behaviors of inactive adolescents, particularly from the perspective of PE teachers. Although other school staff including headteachers/principals (20) have an impact, PE teachers are the gatekeepers for delivering interventions in schools and have a better understanding of the complex range of needs, attitudes, and preferences of inactive adolescents since they work with them every week.

In order to effectively change behavior, interventions should be based on evidence and informed by theoretical frameworks. One such framework is the Capability, Opportunity, Motivation and Behavior (COM-B) model, which is widely used in the field of health and specifically in some PA interventions (21). The model recognizes that behavior is part of a complex interacting system that is influenced by an individual’s capability, opportunity, and ‘motivation’ to perform a given behavior (22). Therefore, to encourage a new behavior one or more of these components need to be changed. By using the Behavior Change Wheel, which is a synthesis of 19 different frameworks of behavior change, the model can be applied to identify options for suitable interventions designed to change one or more of the components (i.e., capability, opportunity, and motivation). The hub of the wheel represents the sources of the behavior that could be targeted through interventions and this will be assessed alongside the findings of the study to identify behaviors than can be targeted through intervention to support inactive adolescents. The wheel also includes a layer of nine intervention functions that can be used to modify specific behaviors, as well as seven types of policy that can be employed to deliver the intervention(s).

The Theoretical Domains Framework (TDF) consists of 14 domains (23) and is often used in conjunction with the COM-B model to identify and describe the factors, which may influence a specific behavior. These factors can be mapped onto the COM-B model to identify factors that influence behavior and highlight intervention functions, policy categories and behavior change tools that can inform future intervention design. Finally, to assess the suitability of an intervention for adolescents the APEASE (affordability, practicability, effectiveness, acceptability, safety, and equity) criteria should be applied (22).

The hypothesis for this study is that PE teachers’ unique perspective can provide evidence of barriers faced by inactive groups participating in sports and PA in secondary schools and identify sources of behavior contributing to inactivity. The research questions for the study are:

(1). What are the barriers that inactive groups face when participating in sports and PA in secondary schools, according to PE teachers?
(2). How can the COM-B model, Behavior Change Wheel and TDF be used to identify facilitators of change in schools for inactive adolescents to achieve recommended levels of PA?
(3). Based on the APPEASE criteria, how can future intervention support be targeted to improve success in increasing PA levels among inactive groups in secondary schools?

## 2. Methods

### 2.1. Design and data collection methods

The study utilized a qualitative approach to examine the perceptions of PE teachers working with inactive adolescents in deprived secondary schools (aged 11–16). The study was conducted on behalf of the Department of Health and Social Care (DHSC) and the definition of ‘inactivity’ was based on the guidelines set by Sport England. According to Sport England’s Active Lives Children and Young People survey, there are three categories of activity: ‘active’ (i.e., meeting the Chief Medical Officers’ guidelines–an average of 60+ daily minutes), ‘fairly active’ (i.e., an average of 30–59 min per day) and ‘inactive’ (i.e., an average fewer than 30 min per day).

Five members of the research team conducted structured telephone interviews with 156 Heads of PE in schools and individual telephone interviews lasted on average around 30 min. At the beginning of each interview, a definition of ‘inactive’ was provided to ensure consistency across schools and to help PE teachers understand the focus of the study. The questions were developed based on the COM-B model of behavior change and piloted with five PE teachers. The following are the questions that were asked during the interviews:

(1). Have you as a school collected information to understand...

(a) What do people like or dislike about PE and school sports?
(b) Why do some adolescents not engage in PE lessons or school sports?

(2). What do adolescents like about PE or school sports?
(3). What do adolescents dislike about PE or school sports?
(4). Why do some adolescents not engage in PE lessons or school sports?
(5). What strategies do you have in place to support inactive adolescents?

(a) What is working well?
(b) What is not working well?

(6). What is the main change required to encourage ‘inactive’ adolescents to be more active?

### 2.2. Sample and recruitment

The researchers began by selecting an initial sample of 600 schools from an existing database of schools. To be included in the sample, schools had to have an overall proportion of students equal to or greater than 20% eligibility for free school meals. This criterion was used to identify the most deprived schools in England, where there was a higher likelihood of inactive adolescents. From the initial sample of 600 schools a random sub-sample of 250 schools was selected.

The research team then attempted to contact the PE teachers at each of the schools in the initial sub sample a maximum of five times to arrange interviews. Any schools that were unable to schedule an interview were excluded from the sample. Due to teaching commitments, contacting the Heads of PE was challenging, and many did not respond to the email invite. After approximately 2 weeks, the initial list of schools was exhausted and a further sample of 100 schools was randomly selected from the original database. This process was repeated until all 600 schools had been contacted. Throughout the study, ethical guidelines were strictly followed including providing information sheets, obtaining completed consent forms, and explaining participants rights to withdraw from the study. Anonymity was also assured. The Sheffield Hallam University’s Ethics Committee granted approval for the study.

The sample included PE teachers who were 62% male and represented schools from all regions with a higher concentration in the North West and London. It is unclear whether this sample is representative of the gender distribution of PE teachers in England, since there is a lack of data on this matter. As these two regions made up half of the database, it was expected that a higher proportion of schools from these areas would be included in the study. The researchers used Sport England’s Active People Survey to map the participating schools and identify the geographic clusters where the physically inactive are most prevalent (15). The results showed that the research sample was broadly representative of these clusters.

### 2.3. Data analysis (phase 1)–analysis of interviews

Throughout the interviews, the research team used an online survey tool to record the responses of PE teachers. After each interview, the research team checked the data for accuracy and then utilized Quirkos, a qualitative data management and analysis software. The qualitative data that was obtained from the telephone interviews was then deductively coded using thematic analysis (24) and analyzed through the lens of the TDF mapped onto the COM-B model and Behavior Change Wheel. The barriers to PA were then categorized into sub-themes of capability, motivation and/or opportunity. Additionally, preferences were organized into sub-themes and analyzed to understand potential facilitators for students to participate in PA. The aim of Phase 1 was to identify the barriers and to enable the research team to explore diversity in perspective and alternative explanations for the findings, before agreeing on the final list of barriers to be utilized for the next phase of analysis.

### 2.4. Data analysis (phase 2): COM-B, Behavior Change Wheel and TDF

During Phase 2, the researchers conducted a thorough examination of the barriers and categorized them according to their most relevant domain in the TDF that was mapped to the COM-B, utilizing a seven-step process (19). Afterwards the research team identified target behaviors using the COM-B, specifically focusing on steps 2–3. They then proceeded to specify what changes were necessary for each behavior in order to facilitate increased PA in the context of each domain, which was achieved in step 4. The research team then selected pre-defined intervention functions and policy categories using the COM-B, which were later screened for their relevancy. Finally, the research team used the ‘theory and technique tool’ to identify suitable behavior change tools relevant to each behavior. Step 8 should be undertaken by intervention designers to design an appropriate mode for the delivery of the intervention. The resulting comprehensive list of behaviors, intervention functions, policy categories and behavior change tools are presented in Table 1.

**Table 1:** COM-B and TDF.

Sources of behavior (COM-B)	Theoretical domains framework	Barrier/construct	What needs to happen for target behavior to occur	Intervention function(s)	Policy functions	Behavior change tool(s)
**Physical capability (1)** Physical skill, strength, or stamina.	**Physical skills** An ability or proficiency acquired through practice.	Physical ability/competency to participate in PA.	Support adolescents to develop physical competency (i.e., physical literacy) to perform in PE/school sports (particularly in team sports and for adolescents who are obese).	Training Enablement	Guidelines Service provision	Instruction on how to perform the behavior Behavior practice/rehearsal Graded tasks
**Psychological capability (4)** Knowledge or psychological skills, strength, or stamina to engage in the necessary mental processes.	**Behavioral regulation** Anything aimed at managing or changing objectively observed or measured actions	Body image effects adolescents experience of PA.	Support adolescents to overcome negative feelings due to perceived negative body image which is often amplified in PA setting.	Education Training Enablement	Service provision Communication/marketing	Problem-solving Self-monitoring of behaviorInformation about antecedentsBehavior substitution Reduce negative emotions Conserving mental resources
	Memory, attention, and decision processes.	Previous negative experiences of PA.	Support adolescents and identify coping strategies to address previous negative experiences of sport and PA and consequent negative feelings and emotions.	Education Training	Service provision Communication/marketing	Problem-solving Self-monitoring of behaviorInformation about antecedents Behavior substitution Reduce negative emotions Conserving mental resources
	**Knowledge** An awareness of the existence of something.	Adolescents’ knowledge of the benefits of PA.	Improve knowledge and promote the importance of PA for mental well-being to balance sedentary activity interests in gaming and social media (i.e., ‘screen time’).	Education Training Enablement	Communication/Marketing	Instruction on how to perform a behavior Information about health consequences Information about social and environmental consequences
		Parents are not aware of the benefits of PA.	Communicate the benefits of PA to parents/guardians and how they can positively support their child(ren).	Education Environmental restructuring Enablement	Guidelines Communication/marketing	Instruction on how to perform a behavior Information about health consequencesInformation about social and environmental consequences
**Social opportunity (4)** The opportunity afforded by interpersonal influences, social cues and cultural norms that influence the way that we think about things, e.g., the words and concepts that make up our language.	**Social influences** Those interpersonal processes can cause individuals to change their thoughts, feelings, or behaviors.	Adolescents feel alienated during PA sessions.	Challenge behaviors that alienate those who do not appear to fit perceived social norms in the PA setting (particularly adolescents who are obese) to create an inclusive environment.	Education Enablement Modeling Restrictions	Guidelines Communication marketing	Social reward Social comparison Information about others’ approvalSocial support (practical)
		Adolescents being bullied.	Address instances of bullying that occur during PA.	Environmental restructuring Modeling Restrictions	Guidelines Communication marketing	Social comparison Social support (practical)
		Parents need support to help adolescents to be active.	Provide opportunities for parents to gain support to overcome economic and environmental barriers (i.e., safety concerns, financial constraints) to PA.	Environmental restructuring Enablement	Guidelines Communication/marketing	Social comparison Information about others’ approval Social support (practical)
		Adolescents were worried about their appearance during sport/PA.	Support for adolescents to overcome perceptions of their appearance during PA (e.g., PE kit, excessive sweating).	Education Environmental restructuring Modeling	Guidelines Communication/marketing	Social comparison Information about others’ approval Social support (practical)
**Physical opportunity (7)** Opportunity afforded by the environment involving time, resources, locations, cues, and physical ‘affordance.’	**Environmental context and resources** Any circumstance of a person’s situation or environment that discourages or encourages the development of skills and abilities, independence, social competence, and adaptive behavior	Lack of inclusive opportunities for adolescents.	Provide an inclusive mix of PA in school which promotes opportunities that caters to a variety of preferences.	Environmental restructuring Enablement Restrictions	Environment/social planning Guidelines	Social support (practical) Restructuring the physical environment Restructuring the social environment
		Adolescents’ preferences not informing school sport provision.	‘Student voice’–provide and encourage opportunities for adolescents to communicate their preferences around PA.	Environmental restructuring Enablement Restrictions	Environment/social planning Guidelines	Restructuring the physical environment Restructuring the social environment Adding objects to the environment
		Adolescents unable to access facilities outside of school time.	Provide access to school facilities outside of school time.	Environmental restructuring Enablement Restrictions	Environment/social planning Guidelines	Social support (practical) Restructuring the physical environment Restructuring the social environment Adding objects to the environment
		Adolescents are not able to access individual or health promoting activities.	Provide access to opportunities for adolescents to engage in individual activities / health-promoting activities (particularly for girls) while still promoting the benefits of team sports.	Environmental restructuring Enablement	Environment/social planning Guidelines	Social support (practical) Restructuring the physical environment Restructuring the social environment
		Adolescents not able to access PA during busy (i.e., academic) and stressful periods in school.	Provide/maintain access to PA opportunities during busy/potentially stressful periods (e.g., during exams).	Environmental restructuring Enablement Restrictions	Environment/social planning Guidelines	Social support (practical) Restructuring the social environment
		Adolescents not finding a suitable capability level to participate in PA, particularly in team sports.	Provide opportunities that allow adolescents to participate with others of a similar level of capability (particularly in team sports).	Environmental restructuring Enablement	Environment/social planning Guidelines	Social support (practical) Restructuring the physical environment Restructuring the social environment Avoidance/reducing exposure to cues for the behavior Adding objects to the environment
		Adolescents do not find certain activities to be fun.	Provide opportunities that focus more on fun than competition.	Environmental restructuring Enablement	Environment/social planningGuidelines	Social support (practical) Restructuring the physical environment Restructuring the social environmentAvoidance/reducing exposure to cues for the behavior Adding objects to the environment
**Reflective motivation (5)** Reflective processes involve plans (self-conscious intentions) and evaluations (beliefs about what is good and bad).	**Beliefs about capabilities** Acceptance of the truth, reality, or validity about an ability, talent, or facility that a person can put to constructive use.	Adolescents have a lack of confidence in their abilities.	Support adolescents to develop perceived confidence performing sport/PA (particularly in group/team sport environment).	Education Incentivization Persuasion Coercion	Service provision Communication/marketing Environmental/social planning Guidelines	Verbal persuasion about capability Focus on past success Self-talk Graded tasks Behavioral practice/rehearsal Demonstration of the behaviorInstruction on how to perform the behavior Problem-solving
		Adolescents believe they are not capable of being physically active.	Support adolescents to identify as being someone capable of being physically active.	Education Incentivization Persuasion Coercion	Service provision Communication/marketing Environmental/social planning Guidelines	Verbal persuasion about capability Focus on past success Self-talk Graded tasks Behavioral practice/rehearsal Demonstration of the behavior Instruction on how to perform the behavior Problem-solving
	**Beliefs about consequences**	Adolescents have a fear of failure in PA.	Support adolescents to overcome the fear of failure and reduce unwanted feelings.	Education ncentivization Persuasion Coercion	Service provision Communication/marketing Environmental/social planning Guidelines	Information about health consequences Salience of consequences Information about social and environmental consequences Anticipated regret Information about emotional consequences Pros and cons Comparative imagining of future outcomes Material incentive (behavior) Incentive (outcome) Reward (outcome)
		Adolescents feel inferior during competitive sports.	Support adolescents to overcome feelings of inferiority during competition.	Education Incentivization Persuasion Coercion	Service provision Communication/marketing Environmental/social planning Guidelines	
	**Intentions** A conscious decision to perform a behavior or resolve to act in a certain way.	Adolescents not committing to do 60 min of PA per day.	Encourage daily intentions to do at least moderate PA for 60 min in and out of school.	Education Incentivization Persuasion Coercion	Service provision Communication/marketing Guidelines	Goal setting (behavior) Information about health consequencesIncentive (outcome)

#### 2.4.1. Stage 1–understand the behavior

(1). Define the problem in behavioral terms through analysis of the study data
(2). Select the target behavior.
(3). Specify the target behavior.
(4). Identify what needs to change.

#### 2.4.2. Stage 2–identify intervention options

(5). Identify intervention functions.
(6). Identify policy categories.

#### 2.4.3. Stage 3–identify content and implementation options

(7). Identify behavior change techniques using the ‘theory and technique tool’.
(8). Identify the mode of delivery.

### 2.5. Rigor

The investigators ensured the reliability and validity of the analysis, by adopting a team-based approach to data analysis. This involved a group of researchers collaboratively reviewing the data using quality criteria such as credibility, dependability, confirmability, and transferability (24). By including multiple researchers, the team addressed potential individual biases and ensured a rigorous and nuanced understanding of the research topic. Through this approach, the team addressed credibility, dependability, and confirmability by identifying patterns in the data and creating themes that were interrogated and confirmed about the research aims and the COM-B model (25). Furthermore, to enhance transferability, the investigators conducted further analysis by combining the TDF mapped onto the COM-B model, which allowed the findings to be useful in several ways and applicable to other studies.

## 3. Findings and discussion

The study found that various factors impact inactive adolescents’ habits and behaviors, which determine levels of engagement in PE lessons and participation in extra-curricular sports and PA. These habits and behaviors are reported according to adolescents’ motivations, capabilities, and opportunities toward PA within schools.

### 3.1. Motivation

#### 3.1.1. Sport and PA preferences

PE teachers reported considerable variation in their descriptions of students’ sport and PA preferences within their schools. These preferences differed between schools, across genders and among various demographic groups including different ethnicities and cultural backgrounds. The significant variance in preferences underscores the importance of taking an individualized approach to identifying motivations and overcoming barriers to PA. To this end, some schools implemented ‘student voice’ activities to identify sporting preferences, which in some cases were targeted specifically at the inactive.

*Depends on the fad at the time. Particularly interested in cricket due to their cultural background. If you name a sport from A to Z we have tried it at some point. Within this school, they do prefer more traditional sports (PE teacher – 1)*.

PE teachers stated that inactive students tended to favor individual sports and/or fitness-based activities, particularly among girls, whereas those who were more active or ‘sporty’, tended to prefer traditional team sports. PE teachers reported that adolescents appreciated having a range of activities and the opportunity to choose activities in their PE lessons.

*De-motivated, unsure of activities on offer, not enjoying team sports as much. Lethargic. Bad diet. Don't like team sports (PE teacher – 2)*.

*Depending on the student…students that enjoy PE and are naturally more sporty, like the national things. With the less sporty they like alternative activities / less traditional (PE teacher – 3)*.

PE teachers described that inactive students tended not to enjoy PA, with variations reported between schools and among different demographic and ethnic groups. According to PE teachers many adolescents disliked large team games or whole-year group activities, including cross-country and were generally not fond of traditional team sports.

*[They] dislike team traditional sports. Many rather play individual sports, but schools don't have the opportunity to provide these as the curriculum doesn't offer enough time so have to focus on team sports which are on the curriculum (PE teacher – 4)*.

*[The] less active don't like football and the pressure of being involved in team sports as much i.e., picked on for not playing a part in a team game. Girls don't like being in the spotlight once they're a little bit older, they're self-conscious (PE teacher – 5).*

#### 3.1.2. Disengagement

PE teachers’ had varying perceptions when describing the characteristics of inactive adolescents. Generally, inactive adolescents were seen as more likely to be female, overweight, or obese and image (i.e., body and appearance) conscious. Over 40% of teachers described inactive adolescents as being disengaged, not motivated or reluctant to participate in sports or PA. For Key Stage 4 students, academic pressures left little time for PA as coursework and exam revision were prioritized. Some PE teachers described pupils’ disinterest in PE and PA as being due to them prioritizing time to use digital technology. Generally, boys were more interested in computer games, and/or watching TV, while girls were more interested in social media or communicating with friends either in person or online.

*Students that prefer computers and social media are quieter individuals and less co-ordinated, so they are challenged when they do something physical (PE teacher – 6).*

*[The] influence of technology has made a difference to adolescents. 'Why play sport when we can play a game on a computer?'*

PE teachers expressed concern that inactive adolescents often disengage from sports due to negative experiences they experienced in primary school. This may have caused feelings of fear or anxiety, while some students may have been unable to self-regulate during sessions. Teachers described that habits are formed early in life, and that experiences with PE in primary school play a significant role in later participation in secondary school. As the primary school curriculum is heavily focused on team sports, negative experiences with these activities may have contributed to disengagement in secondary school. Such experiences could have had an impact on their physical or psychological capability, affecting their motivation to participate in sports or PA.

*I asked one child the other day why he didn't like PE. He had a bad experience with PE at primary school which he is still carrying with him (PE teacher – 7).*

### 3.2. Capability

PE teachers often described that inactive students had lower physical capability, and that this difference in ability was often magnified in a team sport setting. Some PE teachers also reported that some students disliked participating in PE in mixed-ability groups or mixed-gender groups, as they did not want to be seen participating in front of people who were more capable or of the opposite sex. Consequently, their physical capability influenced their motivation to participate in these types of sessions. Conversely, some teachers reported that more capable adolescents became frustrated when grouped with less able or less motivated students, leading to ill feelings among peers. Some students also disliked having to participate with peers from outside of their friendship groups.

PE teachers reported that some schools have made changes in provision to cater to the needs of inactive students. As a result, there has been a shift toward fitness-based activities including circuit training, Zumba, yoga, and aerobics. Many PE teachers highlighted that these activities had proven successful in engaging inactive students in their school as they require a lower skill level and can be performed individually reducing self-consciousness. Furthermore, alternative activities have been used to help the inactive habituate to PA and improve physical capability.

*Some adolescents prefer team sports as a general rule, and non-mainstream sports become favourite activities i.e., trampolining, learning and improving/progressing quickly whereas they feel miles behind in other sports i.e., football. Non-mainstream sports, are often on a level playing field and that's where their progress and enjoyment come (PE teacher – 8).*

PE teachers reported that many students had lower psychological capability, partly due to their lower physical capability reducing confidence in their abilities to participate effectively. This was more prominent in those students that were either obese or overweight as they might be reluctant to take part due to a fear that they would not be able to perform effectively. This lack of confidence was observed across a range of activities but was more noticeable in team sports because of the competitive nature of such activities, which often required group participation. In team sports, students ability levels could be compared to those of their peers which could further expose their lack of confidence to the whole group.

According to PE teachers some students may find certain PA difficult due to their weight, and not having enough energy, or sufficient mobility. The issue of body image was also a concern raised by PE teachers, which was not exclusive to overweight and obese adolescents. As one PE teacher described, comparing themselves to other more ‘sporty’ or physically capable students could exacerbate feelings of inadequacy among overweight or obese students.

*Mental barriers and fear of taking part and fear of failure are related to the lack of belief that they can do well and fear of what class they may be in. That’s why targeted interventions work best because students may not feel inadequate. Sometimes you want to be able to straight talk to kids – we are not aware of how we engage and have a conversation with obese people and how do we work with parents? (PE teacher – 9)*.

There were reported incidences of bullying of overweight and obese students, and it was suggested that this might be exacerbated in PE activities if students could not perform effectively or were seen as a liability.

*Peer pressure and bullying is a problem. Can feel isolated if they have a bad experience in a team because they are maybe not as good at certain activities. Problems with body image, different changing rooms help (PE teacher – 10).*

*Less pressure on them when doing an individual activity rather than team sports which often creates added pressure (PE teacher – 11).*

Motivations and particular insecurities held by adolescents were reported around both the preparation and action of performing PA. These include concerns about becoming sweaty and the physical effects of exercise, wearing the required PE kit, performing, and being judged by others. As adolescents got older they were described as becoming less interested in sport and PA due to an increase in social pressures and concerns around body image. Social media and peer pressure were mentioned as factors that may influence adolescents and consequently make them more concerned with appearance and image.

Adolescents may face physical and psychological challenges that may limit their ability to perform an activity and affect their confidence, enjoyment, and motivation to continue participating. It was universally accepted by PE teachers, that inactive students need support to develop psychological and physical capability but there are constraints on resources, time and capacity to provide the necessary support.

### 3.3. Opportunity

#### 3.3.1. Curricular and extra-curricular opportunities

PE teachers are eager to expand the range of curricular and extra-curricular opportunities available to students, in order to expose them to a diverse selection of sport and PA. While team sports like dodgeball may be included, they are more likely to include individual, adventure sports and fitness-based activities. PE Teachers have expressed concerns about the current content of both curriculum PE and extra-curricular activity. Some suggested that a lack of choice and/or a focus on team sports in some schools restricts the choices of students that were less engaged and less interested in competitive sports.

*It's the same core of students that engage with everything. We want to inspire others to get involved. We have tried climbing and trampolining away from traditional / team sports (PE Teacher – 12).*

*Finding activities that they enjoy doing, a lot of them don’t like the traditional team sports of football etc, so it’s breaking down some of the barriers that they can enjoy PE because there are lots of different activities that they can do, that’s our main focus at the moment (PE teacher – 13).*

Students should also be able to participate with peers of similar ability. However, this is often a challenge for PE teachers, due to limited staff time, which can make it challenging to organize a variety of extra-curricular activities for all students, let alone provide enhanced support to inactive students.

*Having time to do this as we are quite a small team. Most staff do 4 or 5 times a week after school and we have lots of other whole-school work to do. Difficulty planning sometimes and doing activities outside of school (PE teacher – 14).*

*Our PE Staff have core teaching responsibility, clubs and coaching sessions, plus other duties within the school that teachers have to do. We have little time to do the smaller more focused activities needed to engage students, giving students individual attention and one-to-one time is difficult (PE – Teacher – 15).*

Parental engagement and support were regarded as a key factor influencing adolescents’ participation in PA. According to PE Teachers parents from lower socio-economic groups were also more likely to have low PA levels themselves, and may not fully understand the potential benefits of PA for their children. This lack of understanding can lead to a lack of support or encouragement for their adolescents to engage in PA. Furthermore, the social opportunities presented by parents can influence the motivations of inactive adolescents, which can be affected by negative experiences of PE or sports in the past. Consequently, this may lead to inactive adolescents having less opportunity and interest to participate in PA, outside of school time. Adolescents who have supportive, engaged parents who are willing to both transport and support their child before, during and after sessions are more likely to participate in PA and benefit. Some PE teachers highlighted that however proactive the school was in trying to engage adolescents, if their parents were not supportive of their child, then there was very little that the school could do to support them.

*Some students come from disadvantaged homes with low income so we provide kits so that isn’t a problem. Most students do not engage purely because they don’t like it and their parents do not see it as valuable and do not support them (PE Teacher – 16).*

*When you meet parents, [it is] all ‘I hated PE at school’. Their attitude is already affecting their child regarding fitness and health, the inactive and overweight ones all start at home (PE Teacher – 17).*

The social inequalities which exist in deprived areas are also a barrier to participation in PA. PE teachers reported a range of challenges for adolescents to access sports outside of school due to anti-social behavior, cost and poor transport links which represent wider system failures.

*The area is quite mixed, not a place where parents will want kids out late. There are football clubs/training sessions that run a bit later, but access to them isn’t there – too far away and time an issue…but support is needed for the school students to get them to and from (PE Teacher – 18).*

Teachers reported that a key factor that limits engagement and participation in PA is a lack of opportunities available for adolescents to be active within their local communities. PE teachers commented that in the areas around their schools, there is a lack of facilities such as parks and green spaces, sports centers, and gyms, and a lack of specific sessions or activities aimed at adolescents. They noted a need for more sessions targeted at adolescents, including less competitive options and more non-traditional activities to appeal to the inactive.

*There isn’t anything for adolescents in the area. There is no sports centre, no facilities. There is nothing for them, which is why they either go home and watch TV and play on the computer, or turn to antisocial behaviour, out of boredom. The facilities and the culture of the community need to change (PE Teacher – 19).*

Deprived areas may experience additional constraints when it comes to promoting PA including more pronounced financial constraints, difficulties with transport and safety concerns. Furthermore, the local environment and context often reinforces unhealthy behaviors, such as the prevalence of low cost, nutritionally deficient fast food options that limit access to healthier options.

Some PE teachers were adamant that any external investment for opportunities to participate in sports or PA outside of school should be focused on widening participation in the community.

*Targeted funding to community organisations – needs to be spent on minority sports or groups that don’t engage in traditional mainstream sports, so a shift to some non-traditional sports i.e., judo, dodgeball, rock climbing etc (PE Teacher – 20).*

#### 3.3.2. Pupil preferences

Efforts to combat obesity and promote healthy lifestyles has led to a shift in the type of activities being delivered in these schools. As previously stated, PE teachers have described a growing interest among inactive students, particularly girls in fitness-based PA. Increasingly, gym and fitness sessions were also being described as becoming increasingly popular due to their non-competitive nature, and focus on improving body shape, fitness, and health. PE teachers have observed students making progress in their physical health and fitness through these activities and have highlighted their potential for promoting lifelong physical activity beyond school years. T This trend also promotes individual activities which adolescents can do in their own space in their own time.

*With them being girls, we have to use fitness, gym, and introduce things that we know girls like to do. We recently got a disco ball that we are using with circuit training - turn down the lights, put the disco ball on, and turn up the music. This is the type of stuff we have to do to engage them. And it works! Most of this is for KS4 as they are leaving school soon and we need to get them engaged in these types of fitness so that they have been introduced to this and might join a gym or go to a fitness class as an adult (PE Teacher – 21).*

*They want to be left alone doing something they are confident in which points to individual sports rather than team sports (PE Teacher – 22)*.

A shift has also occurred in schools to promote activities that are deemed to be more fun rather than competitive. PE teachers reported that some inactive students did not like the competitive element of team sports, and as a result, some schools described an increased emphasis on the fun elements of PE, rather than on competition.

*The lunch clubs work well with the younger age groups, they can come and play and choose what they want to do each day. Relaxed environment focused on fun rather than competition (PE Teacher – 23).*

*Going with what the students want rather than what we can provide - non-competitive, don't play against other schools, work together as a group, building their confidence…offer what they want and link that in with the curriculum (PE Teacher -24).*

There is no suggestion that competitive sport is not fun, but many factors including how team sports are delivered, and the preferences and personalities of the inactive, may influence how these adolescents perceive or experience such activities. These assumptions could be further fuelled by the perception that team sports are not suitable forms of activity for the inactive.

### 3.4. Capability, opportunity, motivation, and behavior and TDF analysis

PE teachers identified a total of 21 barriers to adolescents meeting government guidelines for PA. These barriers were classified into categories based on the COM-B model into physical opportunity (7), reflective motivation (5), social opportunity (4), psychological capability (4) and physical capability (1). Table 1 presents these barriers and corresponding facilitators for change with suggested intervention functions, policy functions and behavior change tools that could be incorporated into future behavior change interventions.

Table 2 shows the aggregate total of intervention functions and policy categories defined by the COM-B and Behavior Change Wheel that are required to provide a broad understanding of where behavior change interventions could be targeted in schools. The most recorded intervention functions were ‘enablement’ (14) and ‘environmental restructuring’ (12). For policy categories, the most recorded were ‘guidelines’ (14) and ‘communication/marketing’ (13).

**Table 2:** Total number of intervention and policy categories.

Intervention functions	No.	Description	Policy categories	No.	Description
Coercion	5	Creating an expectation of punishment or cost.	Communication/marketing	13	Using print, electronic, telephonic, or broadcast media.
Education	8	Increasing knowledge and understanding.	Environmental/social planning	7	Designing and/or controlling the physical or social environment.
Enablement	14	Increasing means/ reducing barriers to increase capability (beyond education and training) or opportunity (beyond environmental restructuring).	Guidelines	14	Creating documents that recommend or mandate practice. This includes all changes to service provision.
Environmental restructuring	12	Modifying the physical environment around someone to influence their behavior.	Service provision	7	Delivering a service.
Incentivization	5	Creating an expectation of reward.			
Modeling	3	Providing an example for people to aspire to or imitate.			
Persuasion	4	Using communication to induce positive or negative feelings to stimulate action.			
Training	4	Imparting skills.			
Restrictions	6	Using rules to reduce the opportunity to engage in the target behavior (or to increase the target behavior by reducing the opportunity to engage in competing behaviors).			

## 4. Discussion

This study was conducted following previous studies (5, 16–18) that have identified barriers to PA and contributed to existing knowledge (4, 16) of challenges to promoting PA and enhancing the quality of PE among inactive adolescents in the United Kingdom. A comprehensive perception of PE teachers was provided for 21 barriers to PA among inactive adolescents. It applied a novel approach that utilizes the COM-B model, BCW (22), and TDF (23) to present policy categories, intervention functions and behavior change tools to support future PA intervention design. This provides a structured, systematic, and replicable approach for developing an intervention strategy grounded in theory (23). By using this approach, researchers and practitioners can identify target behaviors for inactive individuals, specify what needs to change, and select the appropriate intervention functions and behavior change tools to facilitate behavior change.

The findings from the study report barriers exist across all categories of the COM-B model in physical opportunity (7), reflective motivation (5), social opportunity (4), psychological capability (4) and physical capability (1). The majority of these barriers (90%) were reported in previous studies as being barriers to PA from the perspective of children and adolescents (17, 19). This shows that the findings are consistent with the views of children and adolescents that participated in these studies. However, the study also reveals omissions and nuances in the barriers revealed from the perspective of PE teachers. For example, PE teachers highlighted ‘bullying’ which is not PA specific but is as an additional barrier that can influence PA behavior. Also, PE teachers noted that inactive adolescents may be more affected by a lack of awareness regarding the benefits of physical activity. This study highlights the barriers that are more significant for inactive adolescents and provides additional context regarding the experiences of the inactive. This offers valuable insights for designing targeted interventions that are tailored to their specific needs. It is important to note that the research team does not recommend a homogenous approach to intervention design, even though inactive adolescents may be more impacted by previous negative experiences of PA, influenced by social media or peers, and inclined toward individual or fitness-based activities. Rather, the findings should serve as a basis for developing multi-component interventions which meet the unique needs of inactive adolescents.

Overall, the study presents evidence of the extensive change required to support inactive adolescents to achieve 60 min of at least moderate intensity PA across the week (10). It details the range of individual and systemic changes necessary for effecting behavior change and identified intervention functions that should inform future public health intervention design. For each barrier, a list of behavior change tools are reported that were informed by the theory and techniques tool presenting evidence-based solutions to each barrier. Intervention designers can use the findings in this study as an extensive reference point to inform the design and evaluation of multi-component interventions, as recommended by a previous study (4) and WHO (9), to improve the success of future school-based PA interventions. Furthermore, the study’s findings could also inform policy which may lead to systemic changes (8) necessary for overcoming the barriers highlighted in this and other studies and contributing to the aims of the WHO Global Action Plan (9) aimed at enhancing support for adolescents.

It is important to prioritize certain intervention functions that have a broad impact on the barriers identified in the study, in order to support behavior change. For example, there is a need for enablement, to enhance the capabilities of students and for environmental restructuring to shift the physical environment in schools to influence pupil behavior. Physical capability was identified as a factor influencing motivation and participation, with some students being reluctant to participle in mixed-ability and mixed-sex groups. Nevertheless, it is essential for the school environment to facilitate development of physical capability, since various social influences (such as bullying and alienation) can exacerbate the challenges that adolescents face in this regard. For instance, negative experiences of sports in primary schools may have contributed to the disengagement of some students in secondary school, with some students developing feelings of fear or anxiety during sessions. Targeted work with inactive adolescents through service providers may help overcome some of the physical and psychological barriers that are more pronounced among this group. Additionally, it is necessary to adjust the physical and social environment in schools to establish guidelines to support positive habits and effectively reach the intended audience. Improved communication and marketing are therefore required to reinforce key messages and promote PA opportunities for adolescents that have a lack of knowledge of the benefits of PA. By adopting such approaches inactive adolescents can gain the same benefits as their more active peers, including improved health outcomes and academic achievement which could enhance their future life chances (1–3).

When exploring the implementation of behavior change techniques it is important to consider the APEASE criteria (22). Usually, this criteria is applied to a specific intervention, but can also be useful in considering the barriers to implementation. When evaluating the practicality and affordability of policy interventions for promoting physical activity, it is important to consider the barriers identified in the literature, such as limited resources, time constraints, and funding limitations (17). In the current environment, particularly in the UK, there is uncertainty as to whether all policy functions can be fully realized. Furthermore, acceptability and effectiveness can pose additional challenges in school settings, where adolescents may resist behavior change, and teachers and school leaders may have competing priorities that make focusing on physical activity a lower priority. Such challenges may contribute to a lack of progress in supporting adolescents and the poor implementation of PA interventions, as previously reported (4). To address these issues, digital exercise interventions have emerged in the past decade as a tool to improve the diet and PA behaviors of adolescents (26). More recently, conversational Artificial Intelligence (AI) driven solutions including conversational agents have emerged, offering personalized support through rule-based or natural language interactions tailored to the needs and requirements of users (27). The use of digital approaches in schools to promote PA was suggested in some studies (4).

To advance future digital exercise interventions, conversational AI may hold promise. By providing personalized and natural language-based interactions, it could offer scalable, multi-component, and individualized support to help overcome the various barriers identified in this study. If properly tested, monitored, and evaluated for effectiveness, conversational AI could offer a low-cost and resource-efficient means to support students. While there have been some studies (26, 27) on digital exercise interventions for adolescents, none have explored how conversational AI can be used to overcome barriers to PA and therefore further research is required to determine how much digital solutions could prove beneficial and how effective evidence-based content can be delivered to young people. Further, these solutions should be co-designed with young people to develop suitable person-centered approaches that provide engaging content for young people that is evidence-based, accessible and ethical.

A limitation of this study is that it is based on the subjective opinions of PE teachers of inactive adolescents during a single interview, which is not informed by demographic characteristics (apart from age), their background, or the context where they work. However, it was important to provide teachers with anonymity, so they were free to share their thoughts and feelings about their pupils openly. Moreover, as schools do not track participation, their perceptions of the activity levels of adolescents may not be entirely accurate. However, they are better placed than most to both identify and provide context on the barriers that exist.

## 5. Conclusion

The study provides a comprehensive understanding of the barriers to PA in secondary schools. Specifically it explores the habits and behaviors of inactive adolescents, from the unique perspective of PE teachers. The study identified 21 barriers to PA, with the majority consistent with previous studies. However, previous studies have not definitively determined which barriers are most pertinent to either inactive or active students, thus impeding the prioritization of effective interventions. This study highlights the barriers that are particularly salient for inactive adolescents, and it identifies previously unreported barriers that offer greater insight into their experiences in secondary schools.

It is recommended to use the findings of this study to design future PA interventions for inactive adolescents. The study utilized the COM-B model, Behavior Change Wheel and TDF to identify facilitators of change in schools, for inactive adolescents to achieve recommended levels of PA. The study provides a comprehensive approach to developing intervention strategies grounded in theory, offering evidence-based solutions to each barrier. This findings of this study provide extensive reference points for future intervention design, which could inform policy and contribute to the objective of enhancing support for inactive adolescents. The research team recommend multi-component interventions that are personalized to the needs of inactive individuals to support the changes necessary to overcome barriers to PA and achieve recommended PA levels. They also recommend the further development of digital exercise interventions, particularly conversational AI which may afford a more personalized, natural language experience to engage adolescents at scale and overcome the various individual barriers to PA revealed in this and other studies.

## Data availability statement

The raw data supporting the conclusions of this article will be made available by the authors, without undue reservation.

## Ethics statement

The studies involving human participants were reviewed and approved by Sheffield Hallam University Ethics Committee. The patients/participants provided their written informed consent to participate in this study.

## Author contributions

RM collected and analyzed data and wrote the final manuscript. LE collected data and wrote the final manuscript. MG collected and analyzed data. KG collected and analyzed data. EF analyzed data and wrote the final manuscript. All authors contributed to the article and approved the submitted version.

## Funding

The study was funding (£90,000) from the Department of Health and Social Care was provided on completion of the formal tender to conduct a wider study to Review the Least Active in Secondary Schools. This study is based on interview data collected during this study. Department of Health and Social Care played no role in the design of the study and collection, analysis, and interpretation of data and in writing the manuscript.

## Conflict of interest

The authors declare that the research was conducted in the absence of any commercial or financial relationships that could be construed as a potential conflict of interest.

## Publisher’s note

All claims expressed in this article are solely those of the authors and do not necessarily represent those of their affiliated organizations, or those of the publisher, the editors and the reviewers. Any product that may be evaluated in this article, or claim that may be made by its manufacturer, is not guaranteed or endorsed by the publisher.
